# Canine parvovirus (CPV) phylogeny is associated with disease severity

**DOI:** 10.1038/s41598-019-47773-6

**Published:** 2019-08-02

**Authors:** Giovanni Franzo, Claudia Maria Tucciarone, Sira Casagrande, Marco Caldin, Martí Cortey, Tommaso Furlanello, Matteo Legnardi, Mattia Cecchinato, Michele Drigo

**Affiliations:** 1 Department of Animal Medicine, Production and Health (MAPS), University of Padua, Viale dell’Università 16, 35020 Legnaro, PD Italy; 2 “San Marco” Private Veterinary Clinic, Via dell’Industria 3, 35030 Veggiano, PD Italy; 3 Departament de Sanitat i d’Anatomia Animals, Universitat Autònoma de Barcelona, 08193 Cerdanyola del Vallès, Spain; 4 “San Marco” Private Veterinary Laboratory, Via dell’Industria 3, 35030 Veggiano, PD Italy

**Keywords:** Statistical methods, Virus-host interactions, Viral infection

## Abstract

After its first identification in 1978, canine parvovirus (CPV) has been recognized all around the world as a major threat for canine population health. This ssDNA virus is characterized by a high substitution rate and several genetic and phenotypic variants emerged over time. Overall, the definition of 3 main antigenic variants was established based on specific amino acid markers located in a precise capsid position. However, the detection of several minor variants and incongruence observed between the antigenic classification and phylogeny have posed doubts on the reliability of this scheme. At the same time, CPV heterogeneity has favored the hypothesis of a differential virulence among variants, although no robust and consistent demonstration has been provided yet. The present study rejects the antigenic variant concept and attempts to evaluate the association between CPV strain phylogeny, reconstructed using the whole information contained in the VP2 coding gene, and several clinical and hemato-biochemical parameters, assessed from 34 CPV infected dogs at admission. By using different statistical approaches, the results of the present study show an association between viral phylogeny and host parameters ascribable to immune system, coagulation profile, acute phase response and, more generally, to the overall picture of the animal response. Particularly, a strong and significant phylogenetic signal was proven for neutrophil count and WBC. Therefore, despite the limited sample size, a relation between viral phylogeny and disease severity has been observed for the first time, suggesting that CPV virulence is an inherited trait. The likely existence of clades with different virulence highlights once more the relevance of intensive epidemiological monitoring and research on CPV evolution to better understand the virulence determinants, their epidemiology and develop adequate countermeasures.

## Introduction

*Carnivore protoparvovirus 1* is a species within the genus *Protoparvovirus* in the family *Parvoviridae* that includes several viruses of remarkable epidemiological relevance for wild and domestic carnivores^1^. Canine parvovirus (CPV) is one of the most relevant and well-studied members of this group because of its clinical relevance for companion animals. As other carnivore parvoviruses, it is characterized by a relatively simple, single stranded, negative linear DNA genome of about 5.12 kb, featured by two main Open Reading Frames (ORFs) translated in 4 proteins through alternative splicing^2,3^. The first ORF encodes non-structural proteins NS1 and NS2, involved in viral replication, while the second ORF encodes the viral capsid constituents VP1 and VP2. An additional protein (VP3) originates after VP2 cleavage mediated by host proteases^4^.

After its origin, likely due to the new host adaptation of a feline parvovirus-like of wild carnivores^5^, CPV rapidly spread worldwide in the dog population^6,7^, causing severe disease outbreaks and high mortality^8^. Since CPV totally relies on the host cell machinery, viral replication requires actively proliferating cells. This feature largely explains the viral cell tropism and pathogenesis. In fact, CPV recognizes intestinal crypts and lymphoid organs as main tissue targets^9^. Therefore, the most common clinical picture is characterized by vomit and diarrhea in association with anorexia, depression and fever. Fluid and protein losses through the gastrointestinal tract can result in severe dehydration and hypovolemic shock. Gastrointestinal signs are accompanied by severe immunosuppression characterized by lymphopenia and eventually panleukopenia, which, together with the breakdown of the intestinal barrier, facilitate the passage of bacteria and/or endotoxins in the blood stream^10^. Septicemia, endotoxemia, systemic inflammation, coagulation disorders and septic shock are therefore commonly present in CPV infected animals and contribute significantly to the disease severity and lethality^11–13^.

Additionally, CPV infects and replicates in myocardiocytes of pups from immunologically naive bitches, causing a typically fatal myocarditis. However, the high population immunity due to extensive vaccination protocols, has substantially decreased the incidence of this sign^14^.

As other ssDNA viruses, CPV displays a high evolutionary rate, which has led to a remarkable variability at both nucleotide and amino acid level^15^. In fact, the original CPV (CPV-2) was rapidly replaced all around the world by new antigenic variants like CPV-2a and CPV-2b that emerged, respectively, in 1979 and 1984^16^. More recently, a “major” antigenic variant, CPV-2c, has been identified and proven widely distributed^5,17,18^. CPV heterogeneity has led to the speculation about the presence of differential immunological and virulence features among strains. However, up to date and except for anecdotal reports, no study has consistently demonstrated an association between antigenic variants and pathogenicity/virulence^5,19–21^.

The current classification of CPV at sub-species level is mainly based on the presence of certain amino acid residues in specific VP2 positions^5,22^. However, this approach has led to the proposal of a multitude of new variants apart from the aforementioned major ones, complicating CPV nomenclature and the understanding of its epidemiology. Additionally, more extensive analyses have demonstrated the limited consistency of associating certain amino acid positions with virulence^23^, as incongruities between these phenotypic markers and phylogeny have frequently been reported^18^. Consequently, the focus on specific amino acid positions could not mirror the actual relationships among strains, hindering the identification of an association between particular viral clades and pathological/virulence features.

The present study attempts to overcome this issue by using different statistical approaches to evaluate the association between CPV strain phylogeny - reconstructed using the whole VP2 gene - and an extensive database of anamnestic, clinical and hemato-biochemical parameters.

## Results

### Animal and viral population features

Thirty-four subjects, 14 females and 20 males, were enrolled in the study provided the availability of 1) a complete clinical record, 2) a complete, high quality VP2 sequence. Half of the animals were properly vaccinated against CPV, while the remaining did not complete the standard vaccination protocol or were not vaccinated at all. Among the vaccinated dogs, 11.7% (2 out of 17) died compared to 29.4% (5 out 17) of the unvaccinated animals. However, no statistically significant association was found between vaccination status and death (p = 0.398). Similarly, no association was detected between animal gender and death occurrence (p = 1). A more detailed description of the included subject features is provided in Supplementary Table 1.

The obtained VP2 sequences showed a nucleotide average raw genetic distance of 0.5% (interval 0–1.31%) and amino acid average raw distance of 0.5% (interval 0–2.1%). Sampled strains were representative of all major antigenic variants (CPV-2 (n = 1), CPV-2a (n = 23), CPV-2b (n = 7) and CPV-2c (n = 3)) based on VP2 amino acid residues 87 and 426 (Supplementary Fig 1). The amino acid frequency for each VP2 position is provided in Supplementary Fig. 2.

### Phylogenesis-trait association

The first principal component axis of pPCA explained 13.16% of the underlying variability. Although it could seem a modest value, this first component significantly outperformed the other ones in explaining the “global” variability along the tree and it was consequently selected for the following analyses.

**Figure 1:**
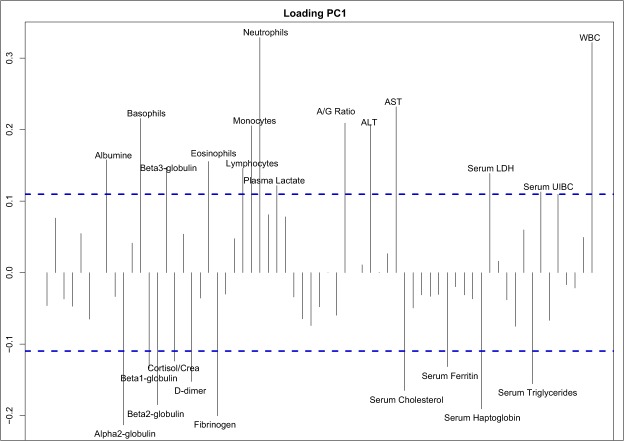
Loadings of the first global component. For interpretation easiness, only the labels of the variables with loadings in the higher 75 percentile (blue dashed lines) are displayed.

PC1 loading evaluation showed the higher contribution to the major global axis of variables ascribable to: immune system functionality (e.g. neutrophils, lymphocytes, monocytes, etc.), coagulation (e.g. D-dimers and fibrinogen), tissue and hepatic enzymes (e.g. serum activity of AST, ALT, LDH), markers of acute phase response (APP) (e.g. α and β globulin, haptoglobin, albumin/globulin ratio (A/G)) and more generally, markers of metabolic derangement (e.g. plasma lactate, cortisol/creatine, cholesterol and triglyceride levels) (Fig. 1).

**Figure 2:**
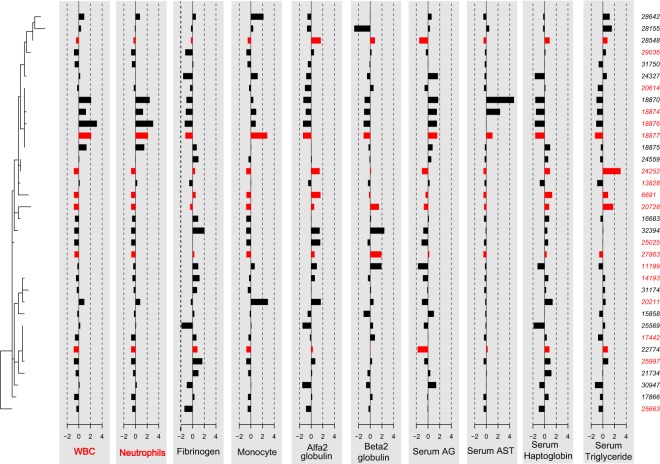
Barplot reporting the standardized values of the hemato-biochemical parameters proven correlated with the viral topology (left insert). Bars of the deceased dogs are colored in red while not properly vaccinated animals are highlighted with red labels. Labels of parameters statistically significant after controlling for False Discovery Rate (FDR) have been marked in red.

Abouheif’s test performed on variables mostly contributing to the first global axis demonstrated a significant phylogenetic signal for: α2 globulin, β2 globulin, monocytes, neutrophils, A/G, serum AST, serum haptoglobin, serum triglycerides and WBC (Table 1 and Fig. 2). However, after controlling for False Discovery Rate (FDR) only neutrophils and WBC remained statistically significant. No statistical association was observed between tree topology and categorical variables like animal gender, vaccination status and disease outcome.

**Table 1:** Summary of statistically associated variables with phylogenetic structure.

	Cmean	p-value
α2-Globulin	0.195	0.030
β2-Globulin	0.144	0.042
Fibrinogen	0.107	0.092
Monocytes	0.128	0.075
Neutrophils*	0.451	0.001
Serum A/G	0.153	0.052
AST	0.083	0.096
Haptoglobin	0.127	0.094
Triglycerides	0.170	0.046
WBC*	0.438	0.001

For each variable the calculated Cmean and the respective p-value are reported.

*parameter statistically significant after controlling for False Discovery Rate (FDR).

No statistical association was observed between tree topology and categorical variables like animal gender, vaccination status and disease outcome. The locus by locus AMOVA results indicate that the nucleotide frequencies in positions 303, 375, 537, 1509 and 1701 differ significantly between the groups defined for each variable. However, all the identified nucleotide variants code for synonymous mutations and no amino acid differences were observed between groups for the considered variables.

## Discussion

The clinical development of infection is an extremely complex phenomenon involving an intricate network of interactions among different concomitant conditions, which are traditionally classified as environmental, host-related and pathogen-related^24^. Among those, the pathogen virulence clearly plays a major role in determining the disease outcome. For several viral species, remarkably different pathological effects and clinical signs have been associated to particular isolates. Differently from more complex organisms, phenotypic features, including virulence, are almost completely genetically determined in microbial pathogens, especially in viruses. Therefore, forms of inheritance from common ancestors, and thus phylogenetic clustering, can be expected. Such association has been speculated for CPV too, and a different virulence among antigenic variants has been hypothesized^19^.

Such association has been speculated for CPV too, and a different virulence among antigenic variants has been hypothesized^19^. Spibey *et al*.^20^ suggested the occurrence of more severe clinical signs in animals infected with CPV-2c. However, these results contrast with previous reports^21^. For instance, Moon *et al*. (2008)^19^ evidenced a higher pathogenicity of CPV-2a compared to CPV-2b Taiwanese strains. Interestingly, the two CPV-2a strains did not show relevant differences in terms of virulence, supporting an actual antigenic variant peculiar virulence. Nevertheless, it is important to note that at the amino acid level the two tested CPV-2a strains (classified based on residue 426 only) differed more within-variant (4 amino acid differences) than between CPV-2a and CPV-2b strains (2 amino acid differences)^19^. Consequently, a proper linkage between viral clustering and clinical consequences is hard to establish. More generally, increasing evidences demonstrate the limits and incongruence of a classification based on phenotypic markers^18^.

Therefore, the present study was designed to investigate the distribution of clinical-pathological data (i.e. outcome of viral infection) along the phylogenetic tree in order to study the CPV genetics-virulence association and consequently its inheritance.

The results of the pPCA indicated that about 13% of the data variability can be explained by first global component, reflecting a relevant tendency of some clinical features to be related to the phylogenetic structure. While the overall variability explained by this axis seems low, it must be stressed that the performed analyses lie on the implicit assumption of considering clinical outcomes as viral features. The actual scenario is clearly much more complex and influenced by other environmental and host factors, as well as the presence of other undetected co-infections and co-occurring diseases, which could not be accounted for in the present study. Consequently, the evidence of a non-negligible pattern between clinical parameters and viral genetics, in spite of this unavoidable “noise”, highlights the relevance of the observed results.

Remarkably, the evaluation of the first global component loadings evidenced that the variables showing a relationship with the viral phylogeny are involved in CPV pathogenesis and associated systemic diseases, leading to multi-organ suffering or failure. A more formal statistical analysis confirmed these results, highlighting the presence of a significant phylogeny signal for several variables that could be globally ascribable to the host response and considered as markers of disease severity (Table 1). Nevertheless, it must be stressed that, after accounting for FDR, only neutrophils and WBC remained statistically significant. Accordingly, a decrease in immune system cell count is a frequent hallmark of CPV infection because of the destruction of myelopoietic cells and several authors have related the lower leukocyte count to a poor prognosis^12,25,26^. While a certain risk of type I error cannot be denied for the remaining parameters (Table 1), they are still worth mentioning because of the concordance of different statistical approaches and the biological plausibility.

Alterations in coagulation system and factor concentrations (e.g. fibrinogen) have been documented in CPV-infected animals^27^, likely because systemic inflammation results in thrombin generation mediated by tissue factor, downregulation of physiological anticoagulant mechanisms, and inhibition of fibrinolysis^28–30^. The hyperfibrinogenemia could also contribute to the pro-coagulative state.

In addition to fibrinogen, other protein levels were identified to be associated with viral genetic features, especially the α2 and β2 globulin components, which comprise elements involved in the acute phase response (i.e. acute phase proteins; APP), like haptoglobin, ceruloplasmin and C-reactive protein. Accordingly, a similar dependency with viral topology was observed in the albumin concentration (negative APP) and A/G ratio. These evidences are particularly suggestive since APPs develop as a precocious response to many pathological stimuli^31^, including infectious agents, leading to amplification of the inflammation and systemic repercussions. An association between APP concentration and disease severity was evidenced for several diseases, including CPV infection^31,32^. Particularly, high serum CRP levels have been positively associated with mortality^33,34^.

Finally, several alterations in hemato-biochemical parameters ascribable to hepatic or renal suffering or variations in the metabolism and acid-base disturbances had an influence on the first global component, albeit the correlation was not always statistically significant. Although these variations are likely not directly caused by CPV, they can be considered a proxy for hypovolemia, shock and hypoperfusion. Alterations in lipid metabolism were also linked to viral phylogeny. Previous studies showed that lipoproteins play a central role during sepsis, binding and neutralizing bacterial lipopolysaccharides, and cholesterol and triglyceride levels were associated with mortality in human patients with sepsis^35,36^. A similar association was also evidenced in dogs with parvoviral enteritis^37^.

All the detected parameters, with few exceptions, can hardly be justified by a direct CPV effect on a particular target, and appear to represent more realistically the overall picture of the animal response.

Unfortunately, the limited sample size and the need to preliminary explore a large number of variables impose a trade-off between type I and II error that hinders and complicates the identification of an association between viral genotype and specific phenotypic consequences on the host, being neutrophils and WBC the most noticeable exception.

Nevertheless, taken as a whole, these results demonstrate a certain relation between viral phylogeny and disease severity, suggesting that CPV virulence is an inherited trait that, originating from an ancestral strain, can characterize descendant clusters.

Quite surprisingly, this evidence was not mirrored by a corresponding clustering of the fatality events in the phylogenetic tree (Figure 2). Likely, many non-viral factors could have affected the disease outcome. Among the others, it must be stressed that, while the clinical and hemato-biochemical parameters were collected at admission, setting a baseline as standard as possible, the following therapeutic approach was modulated case-by-case according to the animal clinical situation and owners’ economic constraints and sympathy. While unavoidable from a practical perspective, this choice surely complicated the interpretation of the relations between viral genetic features and clinical outcome.

The AMOVA analysis evidenced the presence of some nucleotide positions potentially associated with alteration of hemato-biochemical parameters. The absence of corresponding amino acid substitutions could suggest a role of the genome itself in affecting viral biological properties (e.g. secondary structures, codon bias, etc.)^38^ or a linkage of these positions with non-synonymous mutations in other genome regions (i.e. non-structural protein).

Unfortunately, the small sample size due to the difficulty in enrolling a high animal number with associated accurate and standardized hemato-biochemical profiles and reliable viral genetic data limited the statistical power of the study, hindering the detection of the potential association.

Larger studies based on standardized protocols and sharing of the related information among different research groups would surely provide more consistent confirmation of the linkage between CPV phylogeny and virulence by both increasing the study power and exploring a broader viral genetic heterogeneity.

Despite these limitations, the present study suggests, for the first time, the presence of an association between CPV genetic clusters and disease severity, highlighting the inheritableness of this feature. The likely existence of clades with different virulence emphasizes once more the relevance of intensive epidemiological monitoring and research on CPV evolution to better understand the virulence determinants, their epidemiology and arrange adequate countermeasures.

## Material and Methods

### Animals

Archive serum samples collected over the period 2008–2015 from 34  dogs, naturally infected with CPV, were included in the study. Samples originated from San Marco Veterinary Clinic (Padua, Italy) routine clinical activity and were “a posteriori” selected based on the clinical record evaluation. Particularly, stored sera were further analyzed if the corresponding animals showed typical signs of canine parvoviral enteritis and diagnosis was confirmed by CPV specific real-time PCR^18^. Only animals reported to have experienced acute and recent episodes of diarrhea were considered. To limit the effect of other concurrent diseases and co-infections, subjects were included in the study only if no previous and unrelated clinical conditions were reported and blood and/or feces samples tested negative to other common enteric viral, bacterial and parasitic pathogens.

At admission, anamnestic data had been obtained and physical examination performed. Blood samples had been collected and analyzed within the day to investigate several hematological and biochemical parameters (a full list of the investigated variables is provided in Appendix). All animals had been treated with standard supportive therapy until recovery or death. Because of the acute disease nature, the negative outcome (i.e. death) was attributed to CPV infection when occurring within 20 days from hospitalization. Variations from the standard therapy were present due to specific animal clinical conditions (CPV infection course and concomitant diseases) and owners’ compliance. All considered analyses and medical procedures were performed in the context of routine diagnostic and clinical activity and no experimental treatments or additional assays were implemented during the study. Therefore, no ethical approval was required to use specimens collected for diagnostic purpose.

### CPV VP2 characterization and phylogenetic analysis

Archive serum samples were stored at −20 °C from collection to processing. Two-hundred microliters of serum were extracted using NucleoSpin®Blood extraction kit (MACHEREY-NAGEL) and the full VP2 region was amplified with Thermo Scientific™ Phusion™ Hot Start II High-Fidelity DNA Polymerase kit (Life Technologies Inc., Carlsbad, California) with the primers VP-F 5-TTGGCGTTACTCACAAAGACGTGC-3 and VP-R 5-ACCAACCACCCACACCATAACAAC-3, described by Perez *et al*.^39^. Amplification and specificity of the bands were verified by electrophoresis on a SYBR Safe stained agarose gel. PCR products were purified by enzymatic method using Applied Biosystems™ CleanSweep™ PCR Purification Reagent kit (Life Technologies Inc., Carlsbad, California) following the manufacturer’s instructions and Sanger sequenced using 4 overlapping primer pairs as described by Tucciarone *et al*. (2018)^18^.

The quality of the obtained chromatograms was visually evaluated using FinchTV (2004–2006 Geospiza Inc) and consensus sequences were produced using ChromasPro (ChromasPro Version 1.5; Technelysium Pty Ltd, South Brisbane, Australia; http://technelysium.com.au/wp/chromaspro/).

Finally, the sequences were aligned at codon level using TranslatorX^40^ and the region corresponding to the full VP2 was trimmed. The presence of adequate phylogenetic information was evaluated by Likelihood mapping analysis using IQ-Tree^41^. A Maximum Likelihood phylogenetic tree was reconstructed using PhyML^42^ selecting as substitution model the one with the lowest Akaike Information Criterion (AIC) calculated using Jmodeltest^43^. The branch support was calculated by performing 1000 bootstrap replicates.

### Association between clinical data and phylogenesis

Clinical and hemato-biochemical continuous variables were matched to the phylogenetic tree tips using the phylobase R library^44^.

Because of the high number of likely correlated variables, an overall preliminary investigation was performed to identify those potentially associated with the phylogenetic structure. In general, phylogenetic autocorrelation is said to occur whenever the values taken by a set of taxa for a given biological trait are not independent from the phylogeny. This non-independence can be positive (i.e. a higher similarity in related taxa than expected by chance, leading to a so called “global structure”) or negative (a higher dissimilarity in related taxa, leading to a “local structure”)^45^. Since our aim was to investigate the presence of cluster-related virulence features, a particular focus was reserved to the analysis of global structures.

To this purpose, a phylogenetic Principal Component Analysis (pPCA) was performed on centered and scaled variables using adephylo^46^, selecting the phylogenetic proximity matrix calculated with Abouheif’s method. This method allows to summarize several traits in a lower number of variables (i.e. Principal Components; PC) exhibiting global or local structures. PCA eigenvalues were evaluated to quantify the amount of variance and phylogenetic correlation expressed by each PC. The first global component was selected and the respective loadings were inspected to evaluate how each trait contributed to the PC. Variables with loading values in the higher quartile (i.e. most contributing variables) were selected for further analyses. The presence of phylogenetic signal was individually evaluated for each of these variables computing the Abouheif’s Cmean using the phyloSignal package^47,48^. The statistical significance of the obtained index was assessed by comparison with a null hypothesis distribution (i.e. absence of phylogenetic signal) obtained performing 1000 repetitions of the test with label randomization. Because of the limited sample number, the statistical significance level was set to p < 0.1. Multiple comparisons were accounted for using the Benjamini-Hochberg procedure to control the False Discovery Rate (FDR) (Benjamini and Hochberg, 1995).

The association between quantitative traits and tree topology was tested calculating different statistics using BaTS: Parsimony score (PI), Association Index (AI) and MC size^49^. To account for the phylogenetic uncertainness, a Bayesian Markov Chain Monte Carlo (MCMC) approach was implemented. A one million generation MrBayes^50^ analysis was performed on CPV alignment sampling model parameters and phylogenetic trees every 5 thousand generations, thus obtaining a tree posterior distribution (after discarding the first 20% of the trees as burn-in). The aforementioned statistics were calculated across these trees, obtaining their posterior distribution. The observed median (μ_0bs_) value was selected as final outcome. The distribution under the null hypothesis (i.e. no trait-phylogenesis association) was obtained by randomizing without replacement the tip-trait association one thousand times for each tree of the posterior distribution. Each randomized dataset was used to calculate the statistics medians (μ_null_), which formed the null distribution. This distribution was used to achieve a p-value by simply evaluating the proportion of simulated values more extreme than the observed one.

### Locus by locus AMOVA analysis

A locus by locus analysis of molecular variance (AMOVA) was performed using the program Arlequin v3.5^51^. For each of the selected variables, the individuals were categorized in two groups based on the sign (positive or negative) of the standardized values. Then, the average frequencies of each nucleotide per position and group were compared. The positions presenting a Fct value (fixation index among groups) larger than 0.05 were considered significant.

## Supplementary information

Supplementary figure 1

Supplementary figure 2

Supplementary table 1

## Data Availability

All sequences used in the present study have been made available in GenBank (MN104181-MN104214) (Supplementary Table 1).

## References

[1] Cotmore SF (2014). The family Parvoviridae. Arch. Virol..

[2] Cotmore, S. F. & Tattersall, P. Structure and organization of the viral genome. in *Parvoviruses*. *Section B The rugged virion* (eds Kerr, J. R., Cotmore, S. F., Bloom, M. E., Linden, R. M. & Parrish, C. R.) 73–94 (Edward Arnold Ltd, 2006).

[3] Parrish, C. R. Parvoviruses. in *Fenner’s Veterinary Virology* (eds Dubovi, E. J. & Maclachlan, J. N.) 224–235, 10.1016/B978-0-12-375158-4.00007-9 (Elsevier, 2011).

[4] Cotmore SF, Tattersall P (2007). Parvoviral Host Range and Cell Entry Mechanisms. Advances in Virus Research.

[5] Decaro N, Buonavoglia C (2012). Canine parvovirus-A review of epidemiological and diagnostic aspects, with emphasis on type 2c. Veterinary Microbiology.

[6] Appel MJ, Scott FW, Carmichael LE (1979). Isolation and immunisation studies of a canine parco-like virus from dogs with haemorrhagic enteritis. Vet. Rec..

[7] Kelly WR (1978). An enteric disease of dogs reselmbing feline panleucopaenia. Aust. Vet. J..

[8] Carmichael LE (2005). An annotated historical account of canine parvovirus. J. Vet. Med. Ser. B Infect. Dis. Vet. Public Heal..

[9] Bloom, M. E. & Kerr, J. R. Pathogenesis of parvovirus infections. *Parvoviruses* 323–325 (2006).

[10] Goddard A, Leisewitz AL (2010). Canine Parvovirus. Veterinary Clinics of North America - Small Animal Practice.

[11] Ramsey, I. *Infectious Diseases of the Dog and Cat*. *Journal of Small Animal Practice* **49**, (Elsevier Health Sciences, 2008).

[12] Schoeman JP, Goddard A, Leisewitz AL (2013). Biomarkers in canine parvovirus enteritis. N. Z. Vet. J..

[13] Turk J (1990). Coliform septicemia and pulmonary disease associated with canine parvoviral enteritis: 88 cases (1987–1988). J. Am. Vet. Med. Assoc..

[14] Ford J, McEndaffer L, Renshaw R, Molesan A, Kelly K (2017). Parvovirus Infection Is Associated With Myocarditis and Myocardial Fibrosis in Young Dogs. Vet. Pathol..

[15] Shackelton LA, Parrish CR, Truyen U, Holmes EC (2005). High rate of viral evolution associated with the emergence of carnivore parvovirus. Proc. Natl. Acad. Sci. USA.

[16] Truyen U (2006). Evolution of canine parvovirus-A need for new vaccines?. Vet. Microbiol..

[17] Buonavoglia C (2001). Evidence for evolution of canine parvovirus type 2 in Italy. J. Gen. Virol..

[18] Tucciarone Claudia Maria, Franzo Giovanni, Mazzetto Eva, Legnardi Matteo, Caldin Marco, Furlanello Tommaso, Cecchinato Mattia, Drigo Michele (2018). Molecular insight into Italian canine parvovirus heterogeneity and comparison with the worldwide scenario. Infection, Genetics and Evolution.

[19] Moon HS (2008). Comparison of the pathogenicity in three different Korean canine parvovirus 2 (CPV-2) isolates. Vet. Microbiol..

[20] Spibey N, Greenwood NM, Sutton D, Chalmers WSK, Tarpey I (2008). Canine parvovirus type 2 vaccine protects against virulent challenge with type 2c virus. Vet. Microbiol..

[21] Decaro N (2005). Clinical and Virological Findings in Pups Naturally Infected by Canine Parvovirus Type 2 Glu-426 Mutant. J. Vet. Diagnostic Investig..

[22] Martella V, Decaro N, Buonavoglia C (2006). Evolution of CPV-2 and Implicance for Antigenic/Genetic Characterization. Virus Genes.

[23] Markovich JE (2012). Effects of canine parvovirus strain variations on diagnostic test results and clinical management of enteritis in dogs. J. Am. Vet. Med. Assoc..

[24] Scholthof K-BG (2007). The disease triangle: pathogens, the environment and society. Nat. Rev. Microbiol..

[25] Bastan I, Kurtdede A, Özen D (2013). Prognostic usefulness of some parameters in dogs with canine parvovirus. Ankara Üniv Vet Fak Derg.

[26] Goddard A, Leisewitz AL, Christopher MM, Duncan NM, Becker PJ (2008). Prognostic usefulness of blood leukocyte changes in canine parvoviral enteritis. J. Vet. Intern. Med..

[27] Otto CM, Rieser TM, Brooks MB, Russell MW (2000). Evidence of hypercoagulability in dogs with parvoviral enteritis. J. Am. Vet. Med. Assoc..

[28] Levi M, Keller TT, Van Gorp E, Ten Cate H (2003). Infection and inflammation and the coagulation system. Cardiovasc. Res..

[29] Witmer CM (2013). Hematologic manifestations of systemic disease (including iron deficiency, anemia of inflammation and DIC). Pediatr. Clin. North Am..

[30] Margetic S (2012). Inflammation and haemostasis. Biochem. medica.

[31] Ceron JJ, Eckersall PD, Martýnez-Subiela S (2005). Acute phase proteins in dogs and cats: current knowledge and future perspectives. Vet. Clin. Pathol..

[32] Yamamoto S (1993). Changes in serum C-reactive protein levels in dogs with various disorders and surgical traumas. Vet. Res. Commun..

[33] Kocaturk M (2010). Prognostic value of serum acute-phase proteins in dogs with parvoviral enteritis. J. Small Anim. Pract..

[34] McClure V, van Schoor M, Thompson PN, Kjelgaard-Hansen M, Goddard A (2013). Evaluation of the use of serum C-reactive protein concentration to predict outcome in puppies infected with canine parvovirus. J. Am. Vet. Med. Assoc..

[35] Colangiulo A, Angelini L, Gallerani M, Zuliani G, Gazzola K (2017). Serum lipid profile and survival in patients with sepsis. Nutr. Metab. Cardiovasc. Dis..

[36] Lee SH (2015). Prognostic Implications of Serum Lipid Metabolism over Time during Sepsis. Biomed Res. Int..

[37] Yilmaz Z, Senturk S (2007). Characterisation of lipid profiles in dogs with parvoviral enteritis. J. Small Anim. Pract..

[38] Franzo G, Tucciarone CM, Cecchinato M, Drigo M (2017). Canine parvovirus type 2 (CPV-2) and Feline panleukopenia virus (FPV) codon bias analysis reveals a progressive adaptation to the new niche after the host jump. Mol. Phylogenet. Evol..

[39] Pérez R (2014). Phylogenetic and genome-wide deep-sequencing analyses of canine parvovirus reveal co-infection with field variants and emergence of a recent recombinant strain. PLoS One.

[40] Abascal F, Zardoya R, Telford MJ (2010). TranslatorX: Multiple alignment of nucleotide sequences guided by amino acid translations. Nucleic Acids Res..

[41] Trifinopoulos J, Nguyen L-T, von Haeseler A, Minh BQ (2016). W-IQ-TREE: a fast online phylogenetic tool for maximum likelihood analysis. Nucleic Acids Res..

[42] Guindon S (2010). New algorithms and methods to estimate maximum-likelihood phylogenies: Assessing the performance of PhyML 3.0. Syst. Biol..

[43] Darriba D, Taboada GL, Doallo R, Posada D (2012). JModelTest 2: More models, new heuristics and parallel computing. Nat. Methods.

[44] Hackathon, R. *et al*. phylobase: Base package for phylogenetic structures and comparative data. *R Packag*. *version 0*.*6* **5** (2013).

[45] Jombart T, Pavoine S, Devillard S, Pontier D (2010). Putting phylogeny into the analysis of biological traits: A methodological approach. J. Theor. Biol..

[46] Jombart T, Balloux F, Dray S (2010). adephylo: New tools for investigating the phylogenetic signal in biological traits. Bioinformatics.

[47] Abouheif E (1999). A method for testing the assumption of phylogenetic independence in comparative data. Evol. Ecol. Res..

[48] Keck F, Rimet F, Bouchez A, Franc A (2016). Phylosignal: An R package to measure, test, and explore the phylogenetic signal. Ecol. Evol..

[49] Parker J, Rambaut A, Pybus OG (2008). Correlating viral phenotypes with phylogeny: Accounting for phylogenetic uncertainty. Infect. Genet. Evol..

[50] Ronquist F (2012). Mrbayes 3.2: Efficient bayesian phylogenetic inference and model choice across a large model space. Syst. Biol..

[51] Excoffier L, Lischer HEL (2010). Arlequin suite ver 3.5: a new series of programs to perform population genetics analyses under Linux and Windows. Mol. Ecol. Resour..

