# Supplementary material for: Canine parvovirus (CPV) phylogeny is associated with disease severity
Source: Sci Rep. 2019 Aug 2;9:11266. doi: 10.1038/s41598-019-47773-6 (PMC6677720; doi:10.1038/s41598-019-47773-6)
Supplement: Supplementary file 1 — Supplementary figure 1 [file 41598_2019_47773_MOESM1_ESM.pdf]

Canine parvovirus (CPV) phylogeny is associated with disease severity.

Giovanni Franzo[1*], Claudia Maria Tucciarone[1], Sira Casagrande[1], Marco Caldin[2], Martí Cortey[3], Tommaso Furlanello[4], Matteo Legnardi[1], Mattia Cecchinato[1], Michele Drigo[1]

[1]Department of Animal Medicine, Production and Health (MAPS), University of Padua, Viale dell'Università 16, 35020 Legnaro, PD, Italy

[2]"San Marco" Private Veterinary Clinic, Via dell'Industria 3, 35030 Veggiano, PD, Italy

[3]Departament de Sanitat i d'Anatomia Animals, Universitat Autònoma de Barcelona, 08193, Cerdanyola del Vallès, Spain

[4]"San Marco" Private Veterinary Laboratory, Via dell'Industria 3, 35030 Veggiano, PD, Italy

[*]Corresponding author

Supplementary figure 1) Maximum likelihood phylogenetic tree reconstructed based on the VP2 sequence of the strains included in the present study. The labels of strains classified as CPV2, CPV2a, CPV2b and CPV2c have been colored in red, orange, green and blue, respectively. For graphical reasons, only branch support values higher than 70 have been reported.

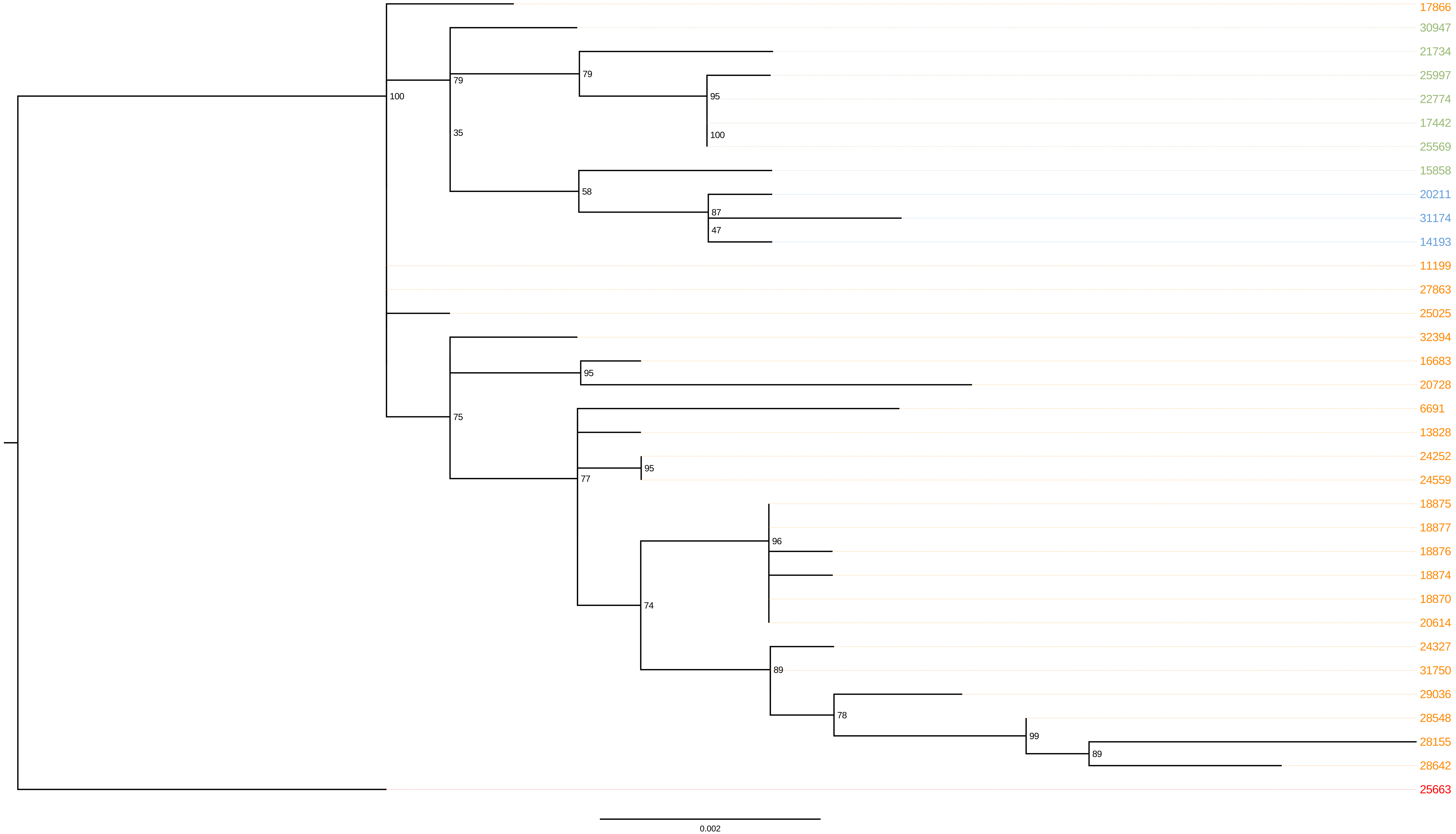
17866
30947
21734
25997
22774
17442
25569
15858
20211
31174
14193
11199
27863
25025
32394
16683
20728
6691
13828
24252
24559
18875
18877
18876
18874
18870
20614
24327
31750
29036
28548
28155
28642
25663
100
79
79
95
100
35
58
87
47
75
95
77
95
96
74
89
78
99
89
0.002
