# Supplementary material for: Canine parvovirus (CPV) phylogeny is associated with disease severity
Source: Sci Rep. 2019 Aug 2;9:11266. doi: 10.1038/s41598-019-47773-6 (PMC6677720; doi:10.1038/s41598-019-47773-6)
Supplement: Supplementary file 2 — Supplementary figure 2 [file 41598_2019_47773_MOESM2_ESM.pdf]

Canine parvovirus (CPV) phylogeny is associated with disease severity.

Giovanni Franzo[1*], Claudia Maria Tucciarone[1], Sira Casagrande[1], Marco Caldin[2], Martí Cortey[3], Tommaso Furlanello[4], Matteo Legnardi[1], Mattia Cecchinato[1], Michele Drigo[1]

[1]Department of Animal Medicine, Production and Health (MAPS), University of Padua, Viale dell'Università 16, 35020 Legnaro, PD, Italy

[2]"San Marco" Private Veterinary Clinic, Via dell'Industria 3, 35030 Veggiano, PD, Italy

[3]Departament de Sanitat i d'Anatomia Animals, Universitat Autònoma de Barcelona, 08193, Cerdanyola del Vallès, Spain

[4]"San Marco" Private Veterinary Laboratory, Via dell'Industria 3, 35030 Veggiano, PD, Italy

[*]Corresponding author

Supplementary figure 2) Logo of amino acid alignment. The overall height of the stack indicates the sequence conservation at that position, while the height of symbols within the stack indicates the relative frequency of each amino or nucleic acid at that position.

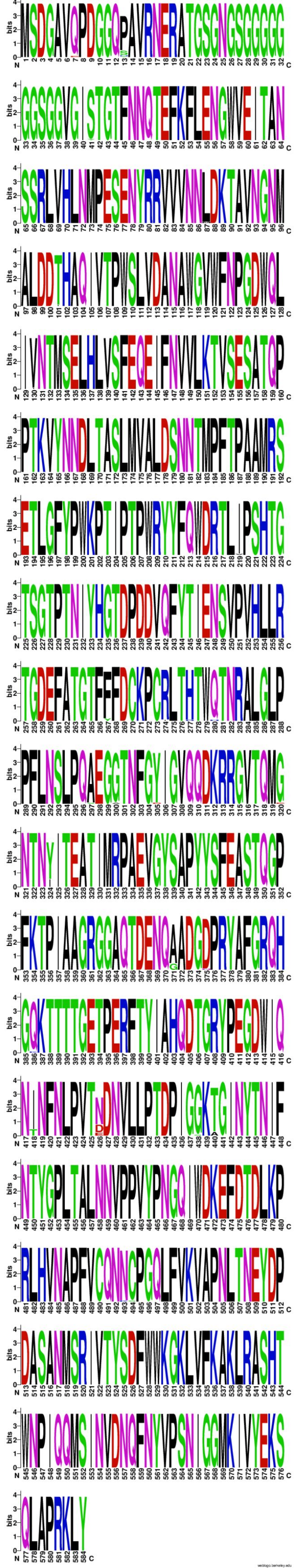
bits
weblogo.berkeley.edu
