# Supplementary material for: Canine parvovirus (CPV) phylogeny is associated with disease severity
Source: Sci Rep. 2019 Aug 2;9:11266. doi: 10.1038/s41598-019-47773-6 (PMC6677720; doi:10.1038/s41598-019-47773-6)
Supplement: Supplementary file 3 — Supplementary table 1 [file 41598_2019_47773_MOESM3_ESM.docx]

**Canine parvovirus (CPV) phylogeny is associated with disease severity.**

Giovanni Franzo^1*^, Claudia Maria Tucciarone^1^, Sira Casagrande^1^, Marco Caldin^2^, Martí Cortey^3^, Tommaso Furlanello^4^, Matteo Legnardi^1^, Mattia Cecchinato^1^, Michele Drigo^1^

^1^Department of Animal Medicine, Production and Health (MAPS), University of Padua, Viale dell'Università 16, 35020 Legnaro, PD, Italy

^2^“San Marco” Private Veterinary Clinic, Via dell'Industria 3, 35030 Veggiano, PD, Italy

^3^Departament de Sanitat i d’Anatomia Animals, Universitat Autònoma de Barcelona, 08193, Cerdanyola del Vallès, Spain

^4^“San Marco” Private Veterinary Laboratory, Via dell'Industria 3, 35030 Veggiano, PD, Italy

^*^Corresponding author

| **aNIMAL ID** | **cpv aCC. nUMBER** | **Collection date** | **Country** | **Host** | **Breed** | **Sex** | **AGE (Months)** | **Vaccination** | **Death** |
| --- | --- | --- | --- | --- | --- | --- | --- | --- | --- |
| **6691** | MN104181 | 2008 | Italy | Canis lupus familiaris | Slovakian Hound | F | 3 | Irregular | YES |
| **11199** | MN104182 | 2010 | Italy | Canis lupus familiaris | Mongrel | F | 5 | Irregular | NO |
| **13828** | MN104183 | 2010 | Italy | Canis lupus familiaris | Akita Inu | M | 3 | Irregular | NO |
| **14193** | MN104184 | 2010 | Italy | Canis lupus familiaris | Mongrel | F | 3 | Irregular | NO |
| **15858** | MN104185 | 2011 | Italy | Canis lupus familiaris | Bernese Mountain Dog | M | 6 | Regular | NO |
| **16683** | MN104186 | 2011 | Italy | Canis lupus familiaris | English Cocker Spaniel | M | 2 | Regular | NO |
| **17442** | MN104187 | 2011 | Italy | Canis lupus familiaris | Weimaraner | M | 5 | Irregular | NO |
| **17866** | MN104188 | 2012 | Italy | Canis lupus familiaris | Dachshund | F | 9 | Regular | NO |
| **18877** | MN104189 | 2012 | Italy | Canis lupus familiaris | Bernese Mountain Dog | M | 2 | Irregular | YES |
| **18876** | MN104190 | 2012 | Italy | Canis lupus familiaris | Bernese Mountain Dog | M | 2 | Irregular | NO |
| **18875** | MN104191 | 2012 | Italy | Canis lupus familiaris | Bernese Mountain Dog | M | 2 | Regular | NO |
| **18874** | MN104192 | 2012 | Italy | Canis lupus familiaris | Bernese Mountain Dog | F | 2 | Irregular | NO |
| **18870** | MN104193 | 2012 | Italy | Canis lupus familiaris | Bernese Mountain Dog | M | 2 | Regular | NO |
| **20211** | MN104194 | 2012 | Italy | Canis lupus familiaris | Mongrel | F | 53 | Unknown | NO |
| **20614** | MN104195 | 2012 | Italy | Canis lupus familiaris | Italian Corso Dog | M | 5 | Irregular | NO |
| **20728** | MN104196 | 2013 | Italy | Canis lupus familiaris | Chihuahua | M | 7 | Irregular | YES |
| **21734** | MN104197 | 2013 | Italy | Canis lupus familiaris | American Staffordshire Terrier | F | 18 | Regular | NO |
| **22774** | MN104198 | 2013 | Italy | Canis lupus familiaris | Rottweiler | F | 8 | Regular | YES |
| **24252** | MN104199 | 2014 | Italy | Canis lupus familiaris | Bichon Frise' | M | 3 | Irregular | YES |
| **24327** | MN104200 | 2014 | Italy | Canis lupus familiaris | Mongrel | F | 3 | Regular | NO |
| **24559** | MN104201 | 2014 | Italy | Canis lupus familiaris | Labrador Retriever | M | 3 | Regular | NO |
| **25025** | MN104202 | 2014 | Italy | Canis lupus familiaris | Maltese | F | 2 | Irregular | NO |
| **25569** | MN104203 | 2014 | Italy | Canis lupus familiaris | Mongrel | M | 3 | Regular | NO |
| **25663** | MN104204 | 2014 | Italy | Canis lupus familiaris | Mongrel | M | 2 | Irregular | NO |
| **25997** | MN104205 | 2014 | Italy | Canis lupus familiaris | Mongrel | F | 3 | Irregular | NO |
| **27863** | MN104206 | 2014 | Italy | Canis lupus familiaris | Chihuahua | F | 5 | Irregular | YES |
| **28155** | MN104207 | 2014 | Italy | Canis lupus familiaris | Maltese | M | 2 | Regular | NO |
| **28548** | MN104208 | 2015 | Italy | Canis lupus familiaris | Maltese | M | 2 | Regular | YES |
| **28642** | MN104209 | 2015 | Italy | Canis lupus familiaris | Dobermann | F | 4 | Regular | NO |
| **29036** | MN104210 | 2015 | Italy | Canis lupus familiaris | Siberian Husky | M | 3 | Irregular | NO |
| **30947** | MN104211 | 2015 | Italy | Canis lupus familiaris | Bolognese | M | 14 | Regular | NO |
| **31174** | MN104212 | 2015 | Italy | Canis lupus familiaris | Golden Retriever | F | 2 | Regular | NO |
| **31750** | MN104213 | 2015 | Italy | Canis lupus familiaris | Mongrel | M | 6 | Regular | NO |
| **32394** | MN104214 | 2015 | Italy | Canis lupus familiaris | French Bulldog | M | 2 | Regular | NO |

**Supplementary table 1)** Metadata associated to the subjects included in the study. The GenBank accession numbers of the detected CPV strain are also reported.
